# Bladder Leiomyoma presenting as urinary retention: A case report

**DOI:** 10.1016/j.eucr.2022.102253

**Published:** 2022-10-07

**Authors:** Jasmine Kashkoush, Alyssa Park

**Affiliations:** Department of Urology, Geisinger Medical Center, Danville, PA, USA

**Keywords:** Leiomyoma, Retention, Female urology

## Abstract

Leiomyoma of the bladder is a rare diagnosis and accounts for 0.43% of all bladder tumors. We herein present a case of a thirty-nine-year-old female diagnosed with bladder leiomyoma presenting with urinary retention. Workup of her urinary retention revealed a midline bladder neck mass. Transurethral resection of her tumor revealed pathology consistent with bladder leiomyoma. Surgical resection is generally the recommended management of this lesion to make a histopathological diagnosis despite low potential of malignant transformation. Although these tumors are benign, they can cause severe obstructive or irritative symptoms. Further research is needed to determine the pathophysiology of these tumors

## 1 Introduction

Leiomyomas are benign smooth muscle neoplasms that can occur in any organ but occur most commonly in the uterus. Leiomyoma of the bladder is a rare diagnosis and accounts for 0.43% of all bladder tumors. Although they are rare, they are the most common benign soft tissue neoplasm of the bladder.^1^ These tumors are known to cause obstructive or irritative symptoms depending on their size and location.^2^ Initial evaluation may include urinalysis, kidney function, cystoscopy, biopsy, upper tract imaging, and urine cytology to determine the extent of tumor and origin. We present a case regarding bladder leiomyoma in a young Caucasian female presenting with urinary retention.

## 2 Case description

The patient is a 39-year-old premenopausal female with a history of multiple urinary tract infections who presented to her primary care physician with urinary retention. She had a six-month history of small volume voids with straining. She had an elevated post void residual and straight catheterized for over 1000 mLs. She was then referred to urology.

She reported a history of overactive bladder symptoms three years prior. She had previously taken anticholinergics but developed recurrent urinary tract infections, suprapubic pain, nocturia, double voiding, and frequency every hour with no gross hematuria. Her post void residual remained elevated at 381 mL. She was started on self-catheterizations and was instructed to stop taking anticholinergics for alleviation of her urinary retention.

**Fig. 1:**
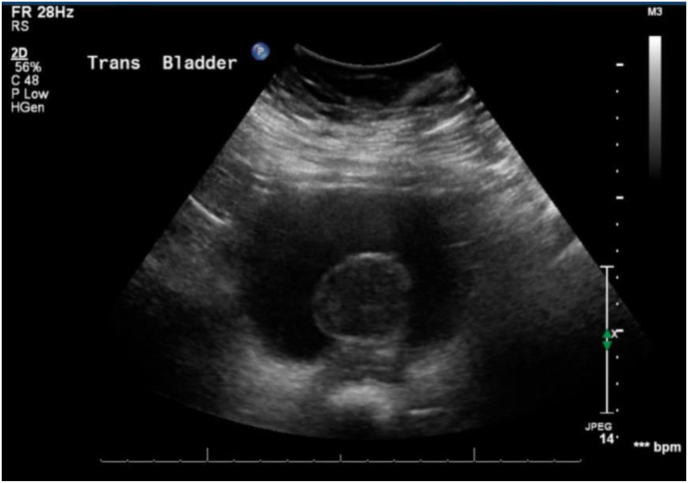
Bladder ultrasonography revealing a solid posterior bladder mass measuring 4.1 × 3.8 × 3.0 cm.

Renal/bladder ultrasonography was significant for a solid posterior bladder mass with vascularity measuring 4.1 × 3.8 × 3.0 cm (Fig. 1). Further evaluation with contrast-enhanced CT confirmed the presence of a posterior bladder mass measuring 3.7 × 3.7 × 4.1 cm, concerning for a transitional cell carcinoma.

**Fig. 2:**
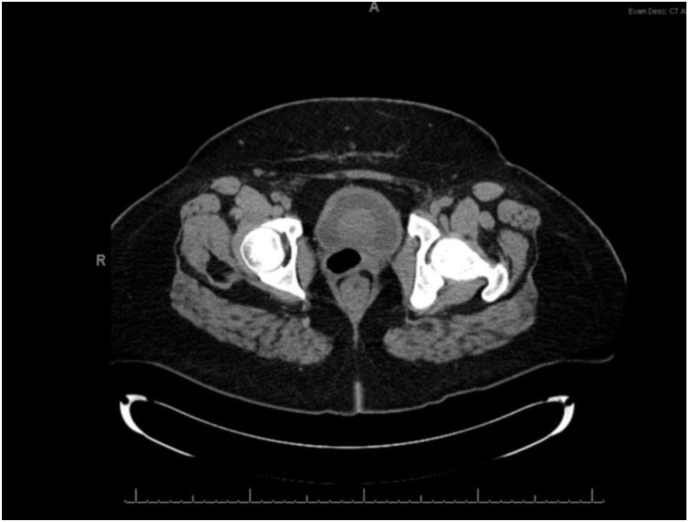
Contrast-enhanced magnetic resonance imaging T2 weighted signal revealing an ovoid smooth mass at the bladder base and neck with hypointense T2 signals, with no transmural extension.

Cystoscopy revealed a well circumcised spherical lesion at the bladder neck with overlying normal appearing urothelium. Bladder cytology did not reveal any malignant or atypical cells. Her urodynamic studies revealed terminal detrusor overactivity with bladder filling and an obstructed pattern with high detrusor pressure (detrusor pressure at peak flow = 124 cm/water) and a low flow rate (average flow rate of 2.1 mL/sec) with voiding. Further evaluation with contrast-enhanced MRI Pelvis revealed an ovoid smooth mass at the bladder neck with heterogeneous isointense T1 and hypointense T2 signals, with no transmural extension (Fig. 2).

**Fig. 3:**
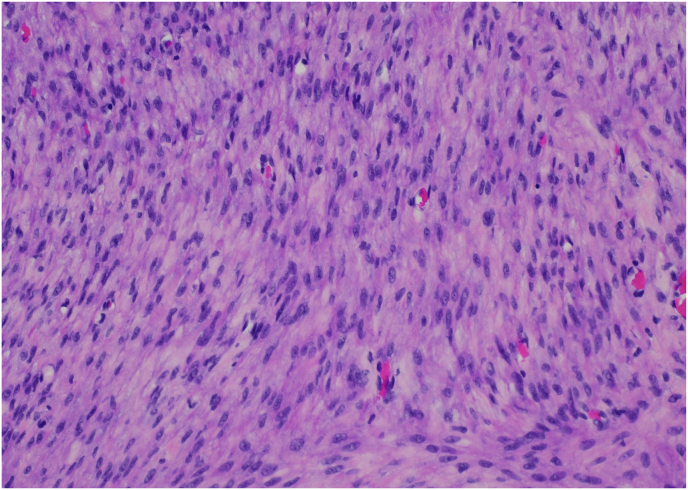
Leiomyoma demonstrating bland spindle cells with no nuclear atypia and no mitosis.

She was taken to the operating room for transurethral resection with pathology revealing a submucosal leiomyoma. Postoperatively, she passed a trial of void. Her only residual symptoms included urgency which was not bothersome enough to start anticholinergic medications. Follow up cystoscopy 1 year post resection revealed a recurrent bladder neck spherical lesion; however, with no urinary retention. She underwent repeat transurethral resection of her 5 cm × 2 cm bladder tumor with pathology revealing a benign leiomyoma (Fig. 3). She returned in 1 year for cystoscopy which revealed a small recurrent bladder leiomyoma at the bladder neck. Her voiding symptoms had improved and thus, she opted out of repeat resection at that time and will return in one year for repeat cystoscopy.

## 3 Discussion

Uterine leiomyomas are the most common benign pelvic tumors in women. Leiomyomas are benign smooth muscle tumors that can be found in any organ. Leiomyoma of the bladder is a rare diagnosis that also occurs more frequently in women.^2^ Although little is known regarding the pathophysiology of these tumors, one of the proposed theories is increasing size secondary to female hormonal influences, given that these tumors tend to occur more commonly in females in their forties when their hormones are most active and there is some evidence that the leiomyomas decrease in size after menopause.^3^^,^^4^ Other proposed theories include growth secondary to dysontogenesis, perivascular inflammation resulting in metaplastic transformation, and infectious and inflammatory sources.

These tumors may present similarly to other bladder tumors as either asymptomatic or with either obstructive or irritative symptoms. Bladder leiomyomas in specific tend to produce symptoms based on their size rather than location.^2^ Larger size masses tend to cause more irritative voiding symptoms including urgency, pain or burning with urination, frequency, or nocturia.^2^ Other reported presentations include hematuria,^1^^,^^2^ flank pain,^1^^,^^2^ and febrile urinary tract infection.

Any bladder lesion requires a thorough evaluation including appropriate imaging and diagnostic evaluation which may include biopsy, cystoscopy, and resection. Initial imaging may include ultrasonography or contrast-enhanced computed tomography. If a leiomyoma is suspected, further imaging with magnetic resonance imaging may be done. Bladder leiomyomas are known to occur most commonly endovesically but may also occur intramurally, and extravesically.^1^^,^^3^ Ultrasound may reveal a smooth, homogeneous, solid mass. Imaging with T2-weighted images as a low signal intensity.^2^

In terms of management, leiomyomas of the bladder are generally treated by surgical resection given the need to make a definitive histopathological diagnosis and given the good prognosis with no evidence of any malignant transformation associated with resection.^2^ On histology, leiomyomas demonstrate bland spindle cells with no nuclear atypia and no mitosis (Fig. 3). There is abundant eosinophilic cytoplasm and oval to cigar-shaped nuclei (Fig. 3). Surgical resection may include transurethral resection, transvaginal excision, or partial cystectomy.^5^

There have been few reports of recurrent bladder leiomyomas that required repeat resection. These seem to be related to a difficult and incomplete resection of the original mass given the challenging location for resection (bladder neck and ureteral orifice) rather than due to a true recurrence.^2^ Our patient's bladder leiomyoma recurred within a year, however, she remained asymptomatic at the time. This is consistent with prior studies showing recurrence at the bladder neck due to a difficult or incomplete resection given the proximity to the urethral sphincter. There is limited literature on the frequency of recurrence of these lesions, but some have shown recurrence within four to six years. Given the paucity of information regarding leiomyoma of the bladder, further research is needed to provide a thorough understanding its natural history and pathophysiology.

## 4 Conclusion

Leiomyoma of the bladder is a rare diagnosis that accounts for <0.5% of all bladder tumors. These tumors tend to have excellent prognosis after surgical resection with no evidence of any malignant transformation. Further research is needed to determine the pathophysiology of these tumors.

## Funding statement

This research received no specific grant from any funding agency in the public, commercial or not-for-profit sectors.

## Contributorship statement

JK and AP were involved in the design and conception of this manuscript. JK performed the literature search. JK compiled the primary manuscript. JK compiled the figures. All authors critically revised the manuscript. All authors have approved the manuscript as it is written.

## Data sharing

All data pertaining to this research article are included within the manuscript as written.

## Declaration of competing interest

The authors have no personal or institutional interest with regards to the authorship and/or publication of this manuscript.
